# Analysis of Electric Bicycle Riders’ Use of Mobile Phones While Riding on Campus

**DOI:** 10.3390/ijerph19105905

**Published:** 2022-05-12

**Authors:** Yanqun Yang, Linwei Wang, Said M. Easa, Xinyi Zheng

**Affiliations:** 1 College of Civil Engineering, Fuzhou University, Fuzhou 350116, China; yangyanqun@fzu.edu.cn (Y.Y.); 200527225@fzu.edu.cn (L.W.); 2 Department of Civil Engineering, Ryerson University, Toronto, ON M5B 2K3, Canada; seasa@ryerson.ca; 3 School of Humanities and Social Sciences, Fuzhou University, Fuzhou 350116, China

**Keywords:** electric bicycles, mobile phones, structural equation model, university student

## Abstract

Based on the theory of rational action (TRA), overconfidence theory (OT), and deterrence theory (DT), this study explores the reasons for mobile phone use by Chinese students riding electronic bicycles (e-bikes) in Fuzhou City. We tested the reliability and validity of an extended TPB, OT and DT questionnaire (with 531 eligible responses) and constructed a structural equation model of mobile phone use behavior while riding e-bikes, based on the improved model. The structural equation model (SEM) is used to evaluate the relationship between the internal factors of mobile phone riding behavior. The results show that the correlation among mobile phone dependence, punishment mechanism, attitude, and controllable operation impacts e-bike riders’ behavior when using mobile phones while riding.

## 1. Introduction

In recent years, the popularity of smartphones in the world has increased. According to the National Bureau of Statistics of China by 2020 [1], China’s total number of telephone users is 1775.98 million, including 1594.1 million mobile phone users, with a penetration rate of 113.9 mobile phones per 100 people and with an average of more than one mobile phone per person. Due to the rapid update and iteration of smartphones, their functions are becoming more and more powerful. Internet use and mobile phone use have become closely intertwined [2]. Because of the convenience and usability of mobile phones [3,4], more and more road users use mobile phones while driving.

Many researchers have studied the use of mobile phones while driving cars. The use of mobile phones while driving can seriously affect driving performance. For example, using mobile phones while driving has a greater impact on the risk of accidents than other in-car activities [5]. If a mobile phone is used during driving, the driver’s potential hazard perception ability will be significantly reduced [6]. Both hands-free and hand-held mobile phones put drivers at a greater risk of accidents [7]. Although using mobile phones while driving harms driving ability, many people still report that they are involved in this behavior [8].

The impact of using mobile phones on drivers also appears in the process of cycling. Different countries have different restrictions on the use of mobile phones in the process of cycling. In Japan, it is not allowed to use mobile phones when riding bicycles [9]. However, in the Netherlands, mobile phones are not allowed when driving a car, but it is a widespread behavior when riding a bicycle [10]. A study in the Netherlands found that teenage cyclists using electronic devices are more likely to have bicycle collisions with young cyclists. However, for middle-aged and elderly cyclists, the use of portable electronic devices is not an essential factor in bicycle collision [11]. In most countries, including Mexico, it is illegal to use mobile phones while riding. Moreover, some scholars have recently studied the prevalence of using mobile phones while riding motorcycles and observed that 0.64% of people use mobile phones while riding motorcycles [12]. In a study in China, a fundamental reason for cyclists to use mobile phones is mobile phone addiction [13].

Since 2000, the popularity of e-bikes in China has been developing rapidly. It has been popularized in Europe and North America in the past decade [14,15,16]. Compared with ordinary bicycles, electric bikes are generally considered to have a larger range of use, faster speed, and better overall performance [17,18,19]. With the rapid development of China’s economy, e-bikes have occupied the non-motor vehicle market rapidly because of their unique advantages. According to the statistics, the number of e-bikes in China is close to 300 million, ranking the first in the world. One of the crucial reasons for the rapid growth of e-bikes is that they have a faster speed than bicycles [20]. The reason for the frequent e-bike accidents in recent years is that other road users underestimate the operating speed of e-bikes [21]. In China, according to the statistics, from 2013 to 2017, 56,200 casualties caused by e-bikes resulted in 8431 deaths, 63,500 people injured, and direct property loss of 111 million yuan [22]. However, due to the economic, urban scale, public transport level, and other reasons, the residents in developed countries mostly use motor vehicles as the primary mode of travel, and non-motor vehicles account for a small proportion. The overall research on e-bikes is not deep enough, and in developing countries, there is no in-depth research on the use of mobile phones when riding e-bikes. A study in Austria found that e-bike users are usually retired, older, and respondents usually have only one car, with low education and income level [23]. Furthermore, in Suzhou, it was observed that 0.4% of e-bike riders used mobile phones at the intersection [24]. The use of mobile phones during riding is related to age, gender, weather, the distance from the city center, the number of lanes, the duration of red lights and the presence of police [25].

Most of the previous research has focused on using mobile phones by car drivers and bicycle riders. In contrast, the research on the behavior of using mobile phones by e-bike riders is lacking, without an in-depth analysis of the reasons. Nevertheless, in an area with a large proportion of e-bike travel, there is no particular system to study and understand the prevalence and psychological causes of e-bike riders using mobile phones, which impacts the safety and future development of e-bikes. This paper aims to explore the reasons why Chinese students use mobile phones when riding electric bicycles.

## 2. Method

### 2.1. Participants

Before the formal investigation, 30 students were selected for interview and pre-survey. The questionnaire was adjusted according to the results of the pre-survey. This study randomly selected 656 university students from a university in Fuzhou, the eastern city of China, to conduct a questionnaire survey. The questionnaire was posted online through Sojump, a popular online survey tool in China. It has the functions of data collection, management and export. We screened out the questionnaires that were missing or filled in incorrectly and were not within the scope of the study, and finally obtained 531 valid questionnaires, and the effective rate was 81%. When the sample number is higher than 10 times the measurement variables, the structural equation model is credible [26]. Therefore, the sample number of this questionnaire can be used for practical data analysis. Among the sample number, 333 participants were male (62.71%), 198 were female (37.29%); the average age of the subjects was 19.94 years (SD = 1.78, age range 16–25 years), involving students in all stages from the university campus. The additional sample characteristics are detailed in Table 1.

### 2.2. Measurements

#### 2.2.1. Demographic Variables and Frequency of Mobile Phone Use

The demographic variables in this study mainly include age, gender, grade, and frequency of mobile phone use. The frequency of mobile phone use is mainly measured using a 6-subscale. The measurement results are divided into the following three categories: high frequency, medium frequency, and low frequency. As users with a low frequency of mobile phone use may interfere with the research results, the structural equation model analysis was carried out only for the groups with medium and high frequencies of mobile phone use.

#### 2.2.2. Mobile Phone Dependence

There are many international questionnaires to measure the degree of mobile phone dependence, but because of the excessive items and to save time, this paper selects a simple version of the Mobile Phone Dependence Questionnaire (MPIQ) [27,28]. MPIQ is based on a one-dimensional eight-item questionnaire, which evaluates the subjects’ cognition and behavior on mobile phones. Each item takes a Likert scale score from 1 (totally inconsistent) to 5 (complete conformity). The higher the score, the higher the dependence on mobile phones. The validity of the questionnaire has been verified [29]. After processing and analyzing the collected data, the third and sixth problems are abandoned in the subsequent model construction.

#### 2.2.3. Measurement of Other Variables

This paper refers to much literature about the structural equation model, integrates the model into car driving and bicycle riding using mobile phones, and based on this, suggests improvements. At the same time, it fully grasps the unique characteristics of mobile phones used by e-bike riders in the process of riding and conducts semi-structured interviews with the students. In addition to the degree of mobile phone dependence, other variables were determined, including attitude, degree of self-confidence, expected regret, social environment assessment, risk perception, road environment, controllable operation, and behavioral tendency. The specific measurement method of the observed variables is mainly based on the e-bike riding environment in the area. The definitions and descriptions of specific variables are shown in Table 2.

### 2.3. Research Hypotheses and Models

In riding e-bikes, the riders mainly rely on their eyes, ears, and other senses to perceive the surrounding traffic environment. Then, they adjust the direction, speed, and balance of the e-bike through the handlebar, handbrake, and throttle to adapt to the current riding environment and complete various riding activities safely and orderly. In general, keeping the stability of the e-bike and real-time understanding of the changes of road traffic environment are considered the main tasks. In contrast, other tasks that affect the main task are attributed to secondary tasks. In riding, using smartphones is a secondary task, which will seriously affect the rider’s perception of the surrounding road environment, reduce the rider’s riding performance, and even lead to traffic accidents.

The structural equation model is a causal model, which can be used to explain the relationship between observed variables and basic constructs in planned behavior theory and the relationship between constructs [30]. The model consists of a measurement model and structural model. The measurement model is used to analyze the relationship between observation variables and potential variables (basic construct), and the structural model is used to analyze the structural relationship between potential variables (basic construct). The structural equation model in this paper is based on the theory of rational action (TRA), overconfidence theory, and deterrence theory.

According to the TRA, people are rational. They will consider the meaning and consequences of their behavior by integrating all kinds of information before making a specific behavior [31]. Therefore, this paper will extract and expand on some latent variables regarding how riders consider the consequences of using mobile phones in riding, including social environment assessment, expected regret, attitude, behavioral tendency, and risk perception. These variables sufficiently reflect the rider’s thinking about using mobile phones during riding and better explain the rider’s mental journey after using mobile phones.

Many cognitive psychology documents believe that people are overconfident, especially over the accuracy of their knowledge. “Overconfidence” refers to an individual’s inaccurate estimation of his or her abilities and excessive optimism about the potential risks in the environment [32]. An important reason for using a mobile phone during riding is the rider’s overconfidence, believing that this behavior will not bring serious consequences. Therefore, this paper extracts and expands on some latent variables regarding the self-overconfidence of riders, including the degree of self-confidence, the road environment, and controllable operation. These variables fully explain the overconfidence behavior of riders, in combination with the road environment and their own riding experience.

According to Law and Economics, because people have the ability to be rational, bad behavior can be deterred. When other conditions remain unchanged and when the expected punishment rises, the bad behavior will decrease. The extent of the reduction may vary from person to person. Deterrence theory holds that the severity and certainty of punishment are two critical factors restricting criminal behavior [33]. A big reason for using mobile phones when riding e-bikes is that there is no corresponding punishment mechanism to deter riders. Therefore, this paper extracts the latent variable punishment mechanism based on the deterrence theory.

Mobile phone dependence is the core variable of the whole model. Only when the rider has a certain degree of dependence on the mobile phone will there be many instances of using the mobile phone while riding. Moreover, mobile phone dependence will bring many adverse effects to riders. Excessive use of mobile phones will cause physical injury to users [34], thus, affecting the whole riding safety.

The occurrence of behavior is usually affected by multiple factors. According to the above analysis, controllable operation, punishment mechanism, mobile phone dependence and attitude are directly related to the use of mobile phone during riding. In order to better explain the behavior of riders using mobile phones, we adopt different theories to explain it. Based on the previous principles and explanations for selecting the latent variables, the rational action and overconfidence theories (combined with the deterrence theory) were used to develop two hypothetical models.

**Hypothesis** **1** **(H1).** *The road environment (RE) has a significant positive impact on the controllable operation (CO).*

**Hypothesis** **2** **(H2).** *The degree of self-confidence (DSC) has a significant positive effect on the controllable operation (CO).*

**Hypothesis** **3** **(H3).** *Controllable operation (CO) has a significant positive effect on behavioral tendency (BT).*

**Hypothesis** **4** **(H4).** *Mobile phone dependence (MPD) has a significant positive effect on behavioral tendency.*

**Hypothesis** **5** **(H5).** *Punishment mechanism (PM) has a significant negative impact on behavioral tendency (BT).*

**Hypothesis** **6** **(H6).** *Mobile phone dependence (MPD) has a significant positive effect on behavioral tendency (BT).*

**Hypothesis** **7** **(H7).** *Social and environmental assessment (SEA) and mobile phone dependence (MPD) interactions.*

**Hypothesis** **8** **(H8).** *Attitude (ATT) has a significant positive effect on behavioral tendency (BT).*

**Hypothesis** **9** **(H9).** *Social and environmental assessment (SEA) positively impacts attitude (ATT).*

**Hypothesis** **10** **(H10).** *Expected regret (ER) has a significant positive effect on attitude (ATT).*

**Hypothesis** **11** **(H11).** *Risk perception (RP) has a significant positive effect on expected regret (ER).*

Among them, MPD, PM, RE, DSC, RP are exogenous variables, which will not be affected by other variables; SEA, ATT, BT, ER, CO are endogenous variables that are affected by any other variable.

### 2.4. Procedure

This research mainly adopts the research method of self-report, including explaining the investigation and the ethical guarantee of the investigation. This set of scales is issued after seeking the informed consent of school leaders and students themselves, using the principle of stratified sampling to fill in the questionnaire. In filling in the questionnaire, students have the entire initiative, can stop filling in the questionnaire at any time, and do not involve any personal privacy information.

### 2.5. Analysis

We tested the validity and reliability of the questionnaire. SPSS25.0 and AMOS22.0 were used to construct the measurement model and structural equation model [35], and the fitting degree of the model was verified. The model was fitted by standard square fitting statistics and comparative fitness index (CFI) [36], non-standard fit index (TLI) [37], and the square root of approximation error (RMSEA) [38]. When CFI and TLI were greater than 0.90 and RMSEA was less than 0.08, the model fitted well [39,40]. Because χ^2^ statistic is very sensitive to sample size, we used χ^2^/DF to evaluate model fitting. If χ^2^/DF is less than 3, it means that the model is acceptable. If χ^2^/DF is less than 4, it also means that the model is acceptable, especially in a large sample size.

## 3. Results

### 3.1. Reliability and Validity Analysis of the Questionnaires

The alpha measure, proposed by Cronbach [41], is a test of questionnaire reliability (internal consistency). It overcomes the shortcomings of the partial half method and is the most commonly used reliability method in social science research. It is generally believed that the alpha coefficient should be higher than 0.7 [42,43].

The dataset was examined for the adequacy of the factor analysis using the Kaiser-Meyer–Olkin (KMO) measure of sampling adequacy and Bartlett’s sphericity test [44,45]. The results of these statistics verify the sampling sufficiency of the dataset. The primary component extraction method was used to test the structural validity of the scale. The KMO measure was much higher than the acceptable level of 0.50. Bartlett’s sphericity test showed that the measurement of the structure value was interdependent [46]. In addition, the factor loading of the items underlying each factor was greater than 0.4; both were higher than the acceptable level of 0.40 [47]. The Cronbach’s alpha value was between 0.714 and 0.928. In addition, the KMO = 0.854 and Bartlett’s test showed a significance level of *p* < 0.01, indicating that the questionnaire was suitable for factor analysis. The convergent validity was determined by composite reliability (CR) and the average variance extracted (AVE). In general, the value of AVE was greater than 0.5 and the value of CR was greater than 0.7. Although the AVE of the MPD variable was slightly less than 0.5, our subsequent modeling deleted some problems, and the final AVE was 0.59. Therefore, the questionnaire set up in this study has good reliability and validity. The statistics and questionnaire test of the observed variables are shown in Table 3.

### 3.2. Descriptive Statistics

According to the survey results, overall, the risk perception level of riders is at a high level (mean = 3.96), indicating that the students’ risk perception for riding e-bikes using mobile phones is evident. In addition, the behavioral tendency (mean = 3.50) and the degree of self-confidence (mean = 3.53) of using mobile phones while riding tend to be positive. The degree of dependence on mobile phones is at a medium level, with a total score of 8–40. The average score of this study group is 23.66, indicating that the degree of dependence on mobile phones is on the high side. It is worth noting that the students do not seem to use mobile phones because of the good or bad road conditions (mean = 2.04). Evidently, the regret of using mobile phones while riding is generally on the high side (mean = 3.10). In addition, the general influence of other variables was relatively low (mean < 3.00). To summarize the results of the questionnaire analysis, it is not common for the students to ride e-bikes and use mobile phones. Most students can effectively control their riding behavior, which may be related to the success of quality education in colleges and universities.

### 3.3. Structural Equation Model

Firstly, we established the structural equation model through AMOS22.0 and verified the fitting degree of the model. In modification indexes, we found that the M.I. of several other pairs, such as e19–e20, is relatively large, so we corrected it. This may be because the final purpose of the questionnaire is to point to human behavior, resulting in residual correlation. These observation variables (such as MPD5 and DSC1) were all established by the riders through the use of mobile phones with the surrounding environment and people, and there are indeed some relationships in essence. At the same time, the problem description of the measurement indicators in the model was similar, so we corrected it. The two structural models were all acceptable after modification (Table 4). The fitting index of model 1 is as follows: χ^2^/DF = 3.587, GFI = 0.902, NFI = 0.913, CFI = 0.936, IFI = 0.936, TLI = 0.926, RMSEA = 0.070. The fitting index of model 2 is as follows: χ^2^/DF = 2.653, GFI = 0.905, NFI = 0.907, CFI = 0.939, IFI = 0.940, TLI = 0.932, RMSEA = 0.056.

After modification, the path coefficients between all the variables were significant at the level of 0.05, and most of the path coefficients were significant at the level of 0.001. Moreover, the model had good fitting indexes and a good fit with the sample data. In addition, the fundamental fitness analysis showed that the error variance was positive. The CR value (decision value) of all the error variance reached a significant level above 0.05. The standard error of the parameters was between 0.021 and 0.110, and there was no large standard error. The above analysis showed that the model was good quality.

### 3.4. Hypothesis Testing

**Figure 1:**
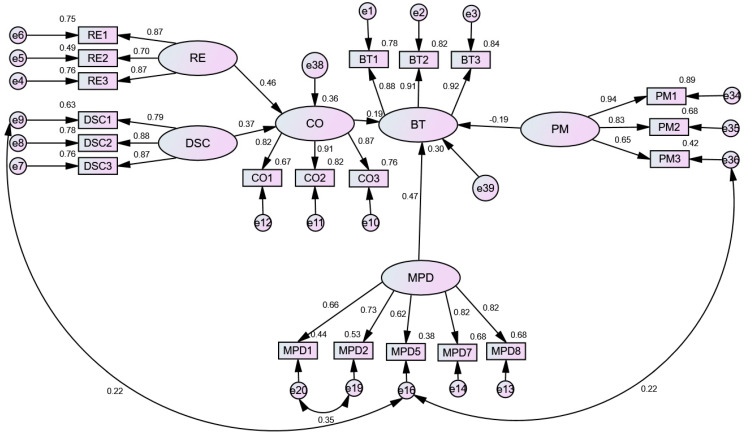
Analysis results of the causes of mobile phone use while riding (Model 1).

**Figure 2:**
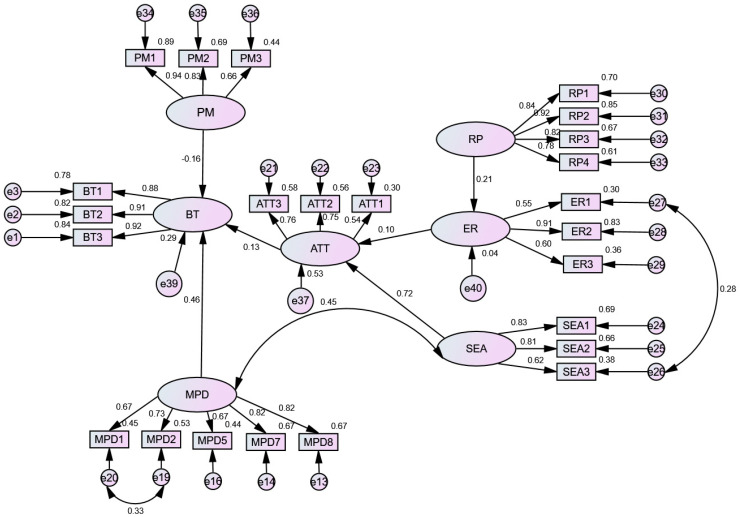
Analysis results of the causes of mobile phone use in riding (Model 2).

The hypothesis relationship was tested by path analysis using the structural equation model. Except for H10 (punishment mechanism adversely impacted the attitude) and H11 (risk perception had a significant negative impact on attitude), H8 and H9 hypotheses were contrary to expectations, and the other hypotheses were accepted. The Figure 1 and Figure 2 show the results of the analysis, including the standardization path. The results of hypothesis testing are summarized as follows:

**Hypothesis** **12** **(H12).** *The road environment (RE) has a significant positive impact on the controllable operation (CO) (β = 0.46, p < 0.001).*

**Hypothesis** **13** **(H13).** *The degree of self-confidence (DSC) has a significant positive effect on controllable operation (CO) (β = 0.37, p < 0.001).*

**Hypothesis** **14** **(H14).** *Controllable operation (CO) has a significant positive effect on behavioral tendency (BT) (β = 0.19, p < 0.001).*

**Hypothesis** **15** **(H15).** *Mobile phone dependence (MPD) has a significant positive effect on behavioral tendency (β = 0.47, p < 0.001).*

**Hypothesis** **16a** **(H16a).** *Punishment mechanism (PM) has a significant negative impact on behavioral tendency (BT) (β = −0.19, p < 0.001).*

The influence degree of the three latent variables (operation controllable, mobile phone dependence, and punishment mechanism in the behavioral tendency level) were 0.19, 0.47, and −0.19, respectively. In particular, mobile phone dependence has a greater impact on behavioral tendency, accounting for 23%, 55%, and 22%, according to the positive normalization.

**Hypothesis** **16b** **(H16b).** *Punishment mechanism (PM) has a significant negative impact on behavioral tendency (BT) (β = −0.16, p < 0.001).*

**Hypothesis** **17** **(H17).** *Mobile phone dependence (MPD) has a significant positive effect on behavioral tendency (BT) (β = 0.46, p < 0.001).*

**Hypothesis** **18** **(H18).** *Social and environmental assessment (SEA) and mobile phone dependence (MPD) interactions. (β = 0.45, p < 0.001).*

**Hypothesis** **19** **(H19).** *Attitude (ATT) has a significant positive effect on behavioral tendency (BT) (β = 0.13, p < 0.05).*

**Hypothesis** **20** **(H20).** *Social environmental assessment (SEA) has a significant positive impact on attitude (ATT) (β = 0.72, p < 0.001).*

**Hypothesis** **21** **(H21).** *Expected regret (ER) has a significant positive effect on attitude (ATT) β = 0.10, p < 0.05).*

**Hypothesis** **22** **(H22).** *Risk perception (RP) has a significant positive effect on expected regret (ER) (β = 0.21, p < 0.001).*

The influence degree of the three latent variables (attitude, mobile phone dependence, and punishment mechanism in behavioral tendency level) were 0.13, 0.46, and −0.19, respectively. Mobile phone dependence has a greater impact on behavioral tendency, accounting for 17%, 24% and 59%, according to the positive normalization.

Overall, mobile phone dependence is a leading factor for riders to use mobile phones when riding e-bikes. The punishment mechanism is one of the relatively essential factors.

## 4. Discussion

In campus traffic, compared with other types of traffic accidents, the safety problem involving e-bikes is persistent and common. On this issue, traffic conditions are different. This is due to the complexity of the road environment of the electric bicycle.

Based on the individual behavior of e-bike riders, this paper has analyzed the psychological factors that affect the use of mobile phones and puts forward some intervention measures. It is vital to analyze the causes of using mobile phones to reduce mobile phone use by e-bike riders during riding. Based on the existing relevant theories, such as the theory of rational action, overconfidence theory, and deterrence theory, this study explores the interaction between various factors to explain the dangerous behavior of using mobile phones while riding. In the sample of Chinese students, we show the consistent relationship between using mobile phones while riding e-bikes and several related factors. In particular, different types of mobile phone use have different correlations with all the results, which indicates that the correlation between mobile phone use and psychological factors may be more subtle than previously thought. The individual characteristics of people are closely related to riding behavior.

### 4.1. Structural Equation Model

This study assumes that controllable operation, mobile phone dependence, and attitude significantly positively correlate with behavioral tendency. Attitude and punishment mechanisms have a significant negative correlation with behavioral tendency. The results show that the improvement of road environment and self-confidence can promote the operation controllable. The operation controllable does have a significant negative correlation with the behavioral tendency. The appearance of the punishment mechanism will reduce the occurrence of behavioral tendency, so it provides support for these hypotheses.

The punishment mechanism has been widely used to study various traffic behaviors [48]. Usually, the appearance of a punishment mechanism will seriously affect one’s view or attitude towards something. However, in this paper, the results show that when using mobile phones when riding, the attitude of the riders seems to have no relationship with this punishment mechanism. In other words, when there is a mechanism to punish the use of mobile phones while riding, it does not change the attitude of riders to continue using the mobile phone. That is, they will continue to maintain such behavior. This may be because of the lack of a corresponding punishment mechanism in reality, and the imaginary mechanism cannot achieve the expected goal. In addition, the correlation between expected regret and social, environmental assessment on attitude is not very consistent with a large number of studies. Generally speaking, when the pressure of the social environment is on a person, their attitude towards something is usually harmful. When the surroundings adversely impact mobile phone use by e-bike riders, it will adversely affect individuals’ attitudes.

One of the crucial reasons for the differences from previous studies may be related to the selection of research objects and research areas. In the campus traffic environment, the pressure of social environment assessment is far less than that of an ordinary road environment, and university students have accepted the use of mobile phones for riding e-bikes as an everyday activity. Therefore, the pressure perception of the surrounding environment is insufficient, and naturally, it will not affect attitude. On the contrary, some students have antagonistic psychology and tend to break the restrictions, so the social environment assessment in this paper has a positive correlation with attitude. The mechanism of regret on attitude is consistent with the above statements. The attitude of riders to traffic safety will impact risk driving behavior [49].

The higher the degree of risk perception of using mobile phones for riding e-bikes, the higher the degree of expected regret for this phenomenon. That is, the frequency of using mobile phones is relatively reduced. This is consistent with previous studies, where people with lower risk perception are more likely to use mobile phones while driving than people with higher risk perception. These findings can predict that the possibility of a person’s participation in risk activities will decrease with the improvement of risk awareness [50,51]. In addition, risk perception can be used to predict behavioral intention [52].

The results also show that the road condition environment and self-confidence have a significant positive correlation with the controllable operation. The road condition environment is more sensitive to the controllable operation. When the road condition environment is more important than self-confidence, it is more critical for the controllable operation. This means that even when a person is not confident enough about himself when the road environment is perfect, the rider will still use mobile phones when riding, which has good research significance for the setting of road conditions. In addition, social environment assessment is related to mobile phone use. On the one hand, when others use mobile phones, they will argue that this behavior is reasonable, and they will follow suit when riding. This is the so-called follow the trend effect [53]. On the other hand, with the powerful function of the mobile phone, it has become a necessity of life, which has fundamentally changed the public’s view of this behavior. As a result, the two factors promote each other, so more and more people use mobile phones when riding.

In higher education institutions, the higher the educational background of the research group, the lower the risk of the behavioral tendency of e-bike riding. This shows that education is a vital factor influencing risk driving behavior tendency [54]. However, some researchers believe that the higher the education level of the drivers, the more they will adopt the corresponding driving style to alleviate the pain caused by anxiety, and the higher the possibility of risky driving behavior [55].

To summarize, a key factor of using mobile phones while riding e-bikes is the mobile phone dependence of riders at present and in the future. The use of mobile phones has become an essential part of students’ lives. When mobile phone dependence is severe, it will cause anxiety and even increase pressure, which will significantly endanger road traffic safety [56]. Although the phenomenon of mobile phone dependence has been recognized as a public health problem by the World Health Organization [57], mobile phones have become an essential tool of social media, which has reached the degree of unavoidable use [58].

Although our research is based on the use of mobile phones when driving cars, drivers usually use mobile phones when driving cars to input and output text messages, listen to phones, navigation and entertainment [59]. These reasons are also applicable to electric bicycles. With the increasingly powerful function of mobile phones, more and more riders are beginning to use mobile phones. With the risk of using mobile phones during driving, some on-board technology equipment has begun to replace the function of mobile phones, and some countries have begun banning the use of mobile phones while driving, essentially controlling mobile phone dependence [60,61]. However, these measures seem difficult to implement on electric bicycles. Based on this discussion, the control of mobile phone dependence is essential, and appropriate measures should be taken to improve this phenomenon.

### 4.2. Recommendations

To reduce traffic accidents of campus e-bikes effectively, this paper, based on the structural equation model constructed above, conducts in-depth research using the following aspects and suggests intervention measures. Given the traffic safety culture under the current administrative policy background, it is a low-cost choice to carry out implicit education in advertising. Advertising needs to match the audience’s needs to achieve high impact, so we explore the causes of using mobile phones while riding e-bikes and develop different forms of traffic safety advertising. In addition, appropriate warning signs can be used to distract riders. These measures may effectively reduce the frequency of mobile phone use [62]. Regarding the traffic policy, given the current lack of punishment mechanism for e-bike riders who use mobile phones, the relevant departments should establish appropriate punishment mechanisms and formulate appropriate traffic rules to control this dangerous traffic behavior.

In order to better solve the problem of college students using mobile phones when riding, we suggest that colleges and universities strengthen training and practice in various ways. These include entrance education, traffic safety courses, educational films on traffic safety warnings, reporting systems for campus traffic accidents, theme-based educational activities, “traffic safety day” publicity, and a visit to an educational workshop. In addition, traffic safety training should be regularly conducted to strengthen traffic safety education and students’ psychological education. Students’ traffic safety consciousness and mental health should be improved from two aspects (daily life and class) to cultivate students’ traffic safety consciousness and fundamentally reduce the use of mobile phone riding behavior.

## 5. Conclusions

Using mobile phones while riding e-bikes does bring some convenience, especially in specific occupational groups. However, it also increases the risk of accidents. Thereby, campus traffic safety authorities must control the use of mobile phones during e-bike riding. This study found that mobile phone dependence, punishment mechanism, and rider’s attitude were the main influencing factors, thus, providing a direction for future research on e-bike traffic safety.

There are still some limitations in the current research. First, the results and models are based on self-reporting, which lacks objectivity. Social expectation is a central problem in the study of self-reported data [63]. It is possible that not everyone will honestly report this type of dangerous behavior. Second, the study participants are only university students, which limits the universality of the research results.

## Figures and Tables

**Table 1:** Sample characteristics.

Observed Variables	*n*	%
Gender		
Male	333	62.71
Female	198	37.29
Education		
Undergraduate	363	68.36
Postgraduate	168	31.64
Riding frequency		
Once a week and less	121	22.79
Several times a week and more	410	77.21
frequency of mobile phone use		
Less than an hour a day	-	-
1–2 h a day	-	-
2–3 h a day	235	44.26
3–4 h a day	122	22.98
4–5 h a day	99	18.64
More than 5 h a day	75	14.12

**Table 2:** Definition and description of latent variables.

Latent Variables	Variable Explanation	Observed Variables
Road environment (RE)	Environment or road condition of using mobile phone by riding	RE1: Good road conditions
		RE2: The waiting time at the intersection is too long
		RE3: Traffic jams during rush hours
Behavioral tendency (BT)	Behavior tendency of using mobile phone while riding	BT1: Passive social media interaction
		BT2: Active social media interaction
		BT3: Usage requirements
Controllable operation (CO)	Riding stability when using mobile phones while riding	CO1: It is still possible to keep balance with your mobile phone while riding
		CO2: Using a mobile phone while riding has no effect on your operation
		CO3: When using mobile phones while riding, you can respond to emergencies in time
Social environment assessment (SEA)	The social environment pressure of riding using mobile phone	SEA1: Conformity behavior
		SEA2: The influence of traffic management countermeasures on the use of mobile
		SEA3: The views of the people around you
Risk perception (RP)	Awareness of the dangers of using mobile phones while riding	RP1: Influence reaction time and reaction degree
		RP2: Lead to distraction
		RP3: Operation deformation
		RP4: Causing traffic accidents
Expected regret (ER)	Regret about using mobile phone while riding	ER1: If you do not use a mobile phone, you will lose the trust of others
		ER2: If everyone else is using a mobile phone in a traffic jam, it is embarrassing to not use it
		ER3: You will miss important opportunities because you do not use your mobile phone to reply to messages
Punishment mechanism (PM)	Using a mobile phone while riding will be punished accordingly	PM1: Fear of being recorded using a mobile phone while riding
		PM2: You are worried that you will be fined if you are found using a mobile phone while riding
		PM3: Worried that using a mobile phone while riding will be announced
Mobile phone dependence (MPD)	Dependence on mobile phones	MPD1: I often think about my mobile phone when I am not using it
		MPD2: I often use my mobile phone for no particular reason
		MPD3: Arguments have arisen with others because of my mobile phone use
		MPD4: I interrupt whatever else I am doing when I am contacted on my mobile phone
		MPD5: I feel connected to others when I use my mobile phone
		MPD6: I lose track of how much I am using my mobile phone
		MPD7: The thought of being without my mobile phone makes me feel distressed
		MPD8: I have been unable to reduce my mobile phone use
The degree of self-confidence (DSC)	Confidence in using mobile phones while riding	DSC1: Can estimate the speed of the E-bike very well
		DSC2: You are confident about your proficiency in riding an E-bike
		DSC3: You can adapt to the changes of the surrounding environment
Attitude (ATT)	Attitude towards using mobile phone while riding	ATT1: Positive attitude; satisfied with this behavior
		ATT2: It does not affect riding
		ATT3: It is an effective use of time and a sense of satisfaction

**Table 3:** Statistics and questionnaire test of the observed variables.

Observed Variables	Mean	SD	Factor Loading	AVE	CR	Cronbach’s Alpha
RE1	1.93	1.04	0.87	0.70	0.88	0.85
RE2	2.16	1.22	0.81			
RE3	2.04	1.16	0.84			
BT1	2.71	1.37	0.82	0.50	0.74	0.73
BT2	2.55	1.20	0.66			
BT3	3.27	1.30	0.61			
CO1	3.30	1.22	0.87	0.73	0.89	0.90
CO2	2.82	1.23	0.85			
CO3	2.82	1.20	0.84			
SEA1	2.97	1.38	0.81	0.60	0.82	0.81
SEA2	3.39	1.32	0.83			
SEA3	2.66	1.33	0.68			
RP1	4.02	0.90	0.88	0.77	0.93	0.91
RP2	3.97	0.90	0.92			
RP3	3.82	0.98	0.85			
RP4	4.01	0.91	0.85			
ER1	2.62	1.07	0.75	0.59	0.81	0.71
ER2	3.05	1.16	0.85			
ER3	3.63	1.11	0.69			
PM1	2.41	1.22	0.84	0.64	0.84	0.85
PM2	2.35	1.24	0.84			
PM3	2.77	1.14	0.72			
MPD1	3.18	1.19	0.77	0.45	0.86	0.87
MPD2	3.27	1.18	0.80			
MPD3	2.50	1.12	0.47			
MPD4	2.89	1.11	0.63			
MPD5	3.23	1.10	0.68			
MPD6	2.32	1.17	0.29			
MPD7	3.12	1.24	0.76			
MPD8	3.15	1.27	0.81			
DSC1	3.49	0.99	0.84	0.73	0.89	0.89
DSC2	3.52	1.06	0.85			
DSC3	3.58	1.01	0.87			
ATT1	3.47	1.10	0.87	0.77	0.91	0.93
ATT2	3.50	1.09	0.89			
ATT3	3.53	1.12	0.86			

**Table 4:** Fit indices of Models 1 and 2.

Index	χ^2^/DF	GFI	CFI	NFI	IFI	TLI	RMSEA
Model 1	3.587	0.902	0.936	0.913	0.936	0.926	0.070
Model 2	2.653	0.905	0.939	0.907	0.940	0.932	0.056

## Data Availability

Not applicable.
